# Does VEGF facilitate local tumor growth and spread into the abdominal cavity by suppressing endothelial cell adhesion, thus increasing vascular peritoneal permeability followed by ascites production in ovarian cancer?

**DOI:** 10.1186/s12943-016-0497-3

**Published:** 2016-02-12

**Authors:** Inga Bekes, Thomas W. P. Friedl, Tanja Köhler, Volker Möbus, Wolfgang Janni, Achim Wöckel, Christine Wulff

**Affiliations:** Department of Obstetrics and Gynecology, University of Ulm, Prittwitzstrasse 42, 89075 Ulm, Germany; Department of Obstetrics and Gynecology, Klinikum Frankfurt Hoechst, Gotenstraße 6-8, 65929 Frankfurt am Main, Germany; Department of Obstetrics and Gynecology, University of Würzburg, Josef-Schneider-Strasse 4, 97080 Würzburg, Germany

**Keywords:** Ovarian cancer, Vascular permeability, Ascites, VEGF, VE-cadherin, Claudin 5

## Abstract

**Background:**

Ovarian cancer is mostly associated with pathologically regulated permeability of peritoneal vessels, leading to ascites. Here, we investigated the molecular regulation of endothelial permeability by the vascular endothelial growth factor (VEGF) and both tight and adherens junction proteins (VE-cadherin and claudin 5) with regards to the tumor biology of different ovarian cancer types.

**Methods:**

Serum and ascites samples before and after surgery, as well as peritoneal biopsies of 68 ovarian cancer patients and 20 healthy controls were collected. In serum and ascites VEGF protein was measured by ELISA. In peritoneal biopsies co-localization of VE-cadherin and claudin 5 was investigated using immunohistochemical dual staining. In addition, the gene expression of VE-cadherin and claudin 5 was quantified by Real-time PCR. Differences in VEGF levels, VE-cadherin and claudin 5 gene expression were analyzed in relation to various tumor characteristics (tumor stage, grading, histological subtypes, resection status after surgery) and then compared to controls. Furthermore, human primary ovarian cancer cells were co-cultured with human umbilical vein endothelial cells (HUVEC) and changes in VE-cadherin and claudin 5 were investigated after VEGF inhibition.

**Results:**

VEGF was significantly increased in tumor patients in comparison to controls and accumulates in ascites. The highest VEGF levels were found in patients diagnosed with advanced tumor stages, with tumors of poor differentiation, or in the group of solid / cystic-solid tumors. Patients with residual tumor after operation showed significantly higher levels of VEGF both before and after surgery as compared to tumor-free resected patients. Results of an immunohistochemical double-staining experiment indicated co-localization of VE-cadherin and claudin 5 in the peritoneal vasculature. Compared to controls, expression of VE-cadherin and claudin 5 was significantly suppressed in peritoneal vessels of tumor patients, but there were no significant differences regarding VE-cadherin and claudin 5 expression in relation to different tumor characteristics. A significant positive correlation was found between VE-cadherin and claudin 5 expression. VEGF inhibition in vitro was associated with significant increase in VE-cadherin and claudin 5.

**Conclusions:**

Our results indicate that increased peritoneal permeability in ovarian cancer is due to down-regulation of adhesion proteins via tumor derived VEGF. Advanced ovarian cancer with aggressive tumor biology may be associated with early dysregulation of vascular permeability leading to ascites. These patients may benefit from therapeutic VEGF inhibition.

## Background

Ovarian cancer is heterogeneous in nature, characterized by differences in tumor growth and survival [1]. Subtypes of more or less aggressiveness are low (LGSOC) and high grade (HGSOC) serous ovarian carcinoma, endometrioid, clear cell and mucinous ovarian cancer [2]. LGSOC is often detected in an early tumor stage and accompanied with well differentiated histology [3]. It may be found incidentally during routine ultrasound or during an operation of another indication [4]. Patients with LGSOC may stay progression free over many years until a new relapse occurs. In contrast, HGSOCs are well known to represent an extremely aggressive, fast-growing cancer usually diagnosed in an advanced tumor stage and carrying poor prognosis compared to other histological subtypes [5, 6]. Often the first clinically noticeable symptom seen in these patients is an increase in abdominal circumference due to high-volume ascites production [7]. The ascites may lead to an intra-abdominal pressure increase with subsequent abdominal pain, nausea, shortness of breath or bowel obstruction [8]. Thus, quality of life of these patients is often extremely limited.

We recently showed for serous papillary ovarian cancer patients that the reason for ascites production is a dysregulated endothelial barrier function of the peritoneal vasculature which leads to an increase of vascular permeability [9]. The endothelial barrier for fluids is built by cell adhesion proteins. Here, adherens junction proteins such as VE-cadherin or tight junction proteins such as claudin 5 seal the space between endothelial cells more or less tightly in dependency of their expression strength. In our recent study, we detected increased levels of the vascular endothelial growth factor/vascular permeability factor (VEGF), a potent cytokine and key regulator of physiological and pathological angiogenesis, in the ascites and serum of ovarian cancer patients associated with a simultaneous decreased amount of the tight junction protein claudin 5 in the peritoneal vessels [9]. In an in vitro model we showed a VEGF-dependent decrease of claudin 5 followed by a significant increase of endothelial permeability, thus ascites production [9]. Besides, a VE-cadherin-dependent expression of claudin 5 in endothelial cells has been reported via the transcription factor FoxO1 [10]. For permeability regulation in the human corpus luteum we previously found that under hCG influence VEGF induces down-regulation of a cascade of adhesion proteins: VEGF dependent down-regulation of VE-cadherin is followed by suppression of other adhesion proteins such as claudin 5, consequently resulting in increased permeability [11].

Given that different ovarian cancer subtypes are associated with variable clinical manifestations and amounts of ascites production, the aim of the present study was to evaluate whether our previously observed mechanism of interaction between VEGF-A_165_ (hereafter named VEGF) - the most important angiogenic factor - and adhesion proteins in serous papillary cancer patients varies depending on histological type and tumor biology. In addition, we focused on the question if under VEGF influence VE-cadherin is interacting with claudin 5 for permeability regulation. More specifically, we investigated VEGF levels in serum and ascites as well as protein and gene expression of VE-cadherin and claudin 5 in peritoneal vessels of ovarian cancer patients in relation to the prognostic factors tumor stage, histological type, tumor grading and resection status after surgery. Moreover, we simulated the in vivo situation for the first time in a co-culture model with human ovarian cancer cells extracted from ovarian cancer patients of our department, and investigated the changes of VE-cadherin and claudin 5 after VEGF inhibition.

## Results

### Patient characteristics

In total 68 patients with ovarian cancer and 20 healthy controls have been collected and analyzed. The patient age ranged from 49 to 77 years for the cancer group and from 49 to 66 years for the controls.

### Histopathologic tumor characteristics

Most ovarian cancer patients presented with serous papillary ovarian cancer (68 %), followed by mixed type tumors (13 %), endometrioid (7 %), solid/cystic-solid (6 %) and mucinous tumors (6 %). 47 % of the patients had an advanced tumor disease (FIGOIIIc). There were 75 % of the ovarian cancer patients with poorly differentiated tumors (G3), 20 % presented with moderately tumor differentiation (G2) and 5 % of the patients had well differentiated tumors (G1). Concerning the postoperative remaining tumor, 55 % of our analyzed patient collective were operated tumor free (TR 0), 28 % had residual tumor left <1 cm and 18 % of the patients had rest tumor >1 cm.

### VEGF-concentration in serum according to time and tumor characteristics

**Fig. 1:**
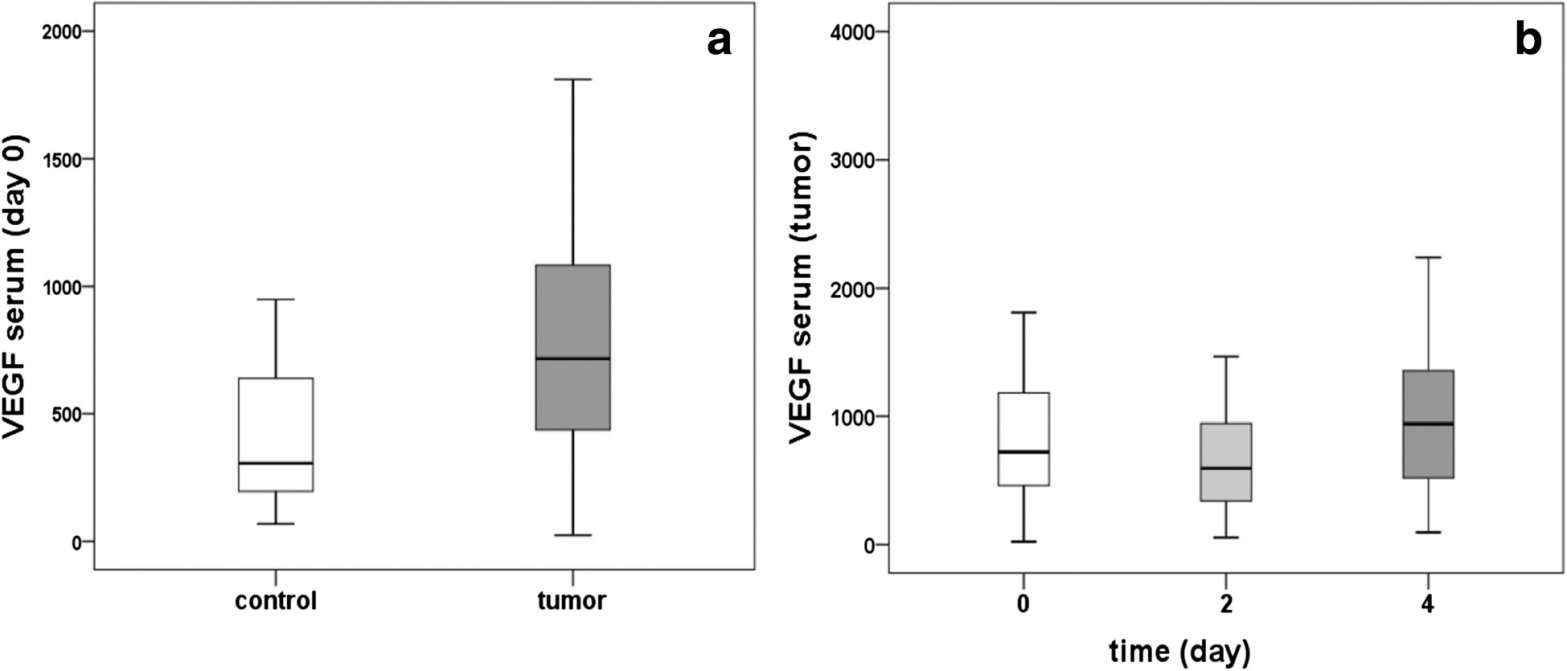
VEGF levels in serum (tumor vs. control) and at different days of measurement (tumor samples). Comparison of VEGF levels (pg/ml) in serum samples between tumor and control group. Statistically significant higher amounts of VEGF levels were detected in the tumor group (*p* = 0.013) (**a**). VEGF levels in serum in the tumor samples before operation (day 0), two days (day 2) and four days (day 4) after surgery. There were statistically significant differences between all days of measurement, with a decrease of VEGF levels at day 2 and increase at day 4 of measurement (all *p* < 0.001) (**b**)

Measurement of VEGF in the serum of ovarian cancer patients on day 0 revealed significant (*p* = 0.013) higher values as compared to healthy controls (Fig. 1a). The analysis of VEGF levels in the serum of ovarian cancer patients at day 2 after surgery revealed a significant (*p* < 0.001) decrease as compared to day 0, while at day 4 after surgery a significant (*p* < 0.001) increase of VEGF levels of tumor patients could be observed (Fig. 1b).

**Fig. 2:**
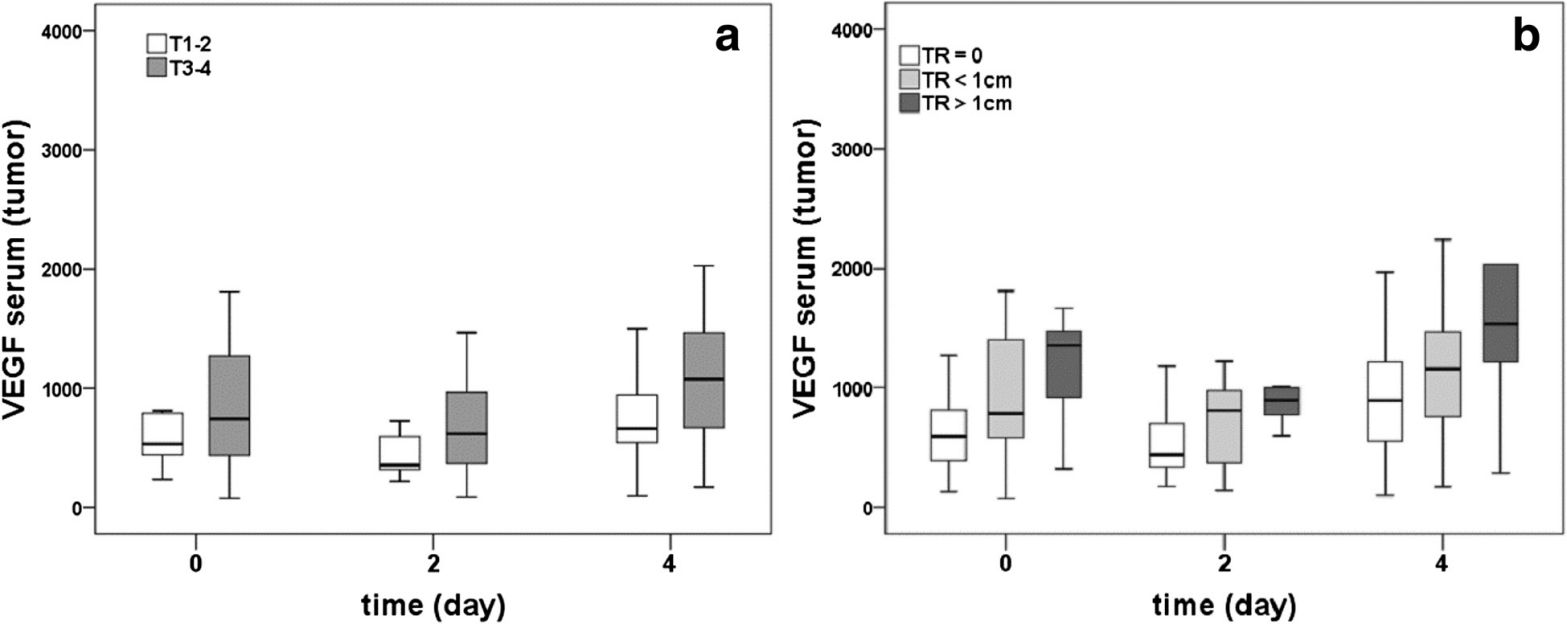
VEGF levels in serum in relation to tumor stage and resection status after surgery. Comparison of VEGF levels (pg/ml) between different groups of tumor sizes (T1-2 vs. T3-4) on the different days of measurement (day 0, day 2 and day 4) showed numerically but not significantly higher values in the group of T3-tumors compared to T1-ovarian cancers (all *p* > 0.07). The differences between the different days of measurement (decrease at day 2 and increase at day 4) were significant in the T3-group (*p* < 0.003) (**a**). Analysis of VEGF levels in the ovarian cancer patient group with no residual tumor after surgery (TR 0) and remaining tumor (TR > 1 cm or TR < 1 cm). We revealed significant higher values in the group with residual tumor (both < and > 1 cm) in comparison to the group with no remaining tumor measured before operation at day 0 (*p* = 0.049 and *p* = 0.003). Significant (all *p* < 0.02) differences between the various days of measurement (0, 2 and 4 days) were detected in all three groups (TR 0, TR < 1 cm, TR > 1 cm), with a decrease in serum VEGF levels at day 2 and an increase on day 4 (**b**)

Patients with larger tumors (T3-4) had higher serum VEGF levels compared to patients with smaller tumors (T1-2) at all days of measurement (day 0, day 2 and day 4; all *p* > 0.07; Fig. 2a). In the group of T3-4 tumors, significant differences in VEGF serum levels could be shown among all three measurement days of measurement, with a significant decrease on day 2 and a significant increase on day 4 (all *p* < 0.003) (Fig. 2a). However, in the group of T1-2 tumors VEGF serum levels differed significantly only between day 0 and day 2 (*p* =0.028) and between day 2 and day 4 (*p* = 0.043).

The analysis of serum VEGF in the ovarian cancer patient group with no residual tumor after surgery (TR 0) and remaining tumor (TR >1 cm or TR <1 cm) revealed that both the group with residual tumor <1 cm and the group with residual tumor >1 cm had significant higher values of serum VEGF at day 0 in comparison to the group with no remaining tumor (*p* = 0,049 and *p* = 0,003, respectively) (Fig. 2b). The highest values were measured in the group with residual tumor left after operation >1cm. Significant (all *p* < 0.02) differences between the various days of measurement (0, 2 and 4 days) were detected in all three groups (TR 0, TR <1 cm, TR >1 cm). (Fig. 2b) with a decrease in serum VEGF at day 2 and an increase on day 4 (Fig. 2b).

The comparison between VEGF values in the serum of different histological types of ovarian cancer revealed no significant differences. Furthermore, no significant differences in the levels of VEGF could be detected between ovarian cancer patients with different tumor grading (G1/G2 vs. G3), different nodal status (N0 vs. N1) or different hormone receptor status (ER/PGR positive vs. negative).

### VEGF-concentration in ascites according to time and tumor characteristics

**Fig. 3:**
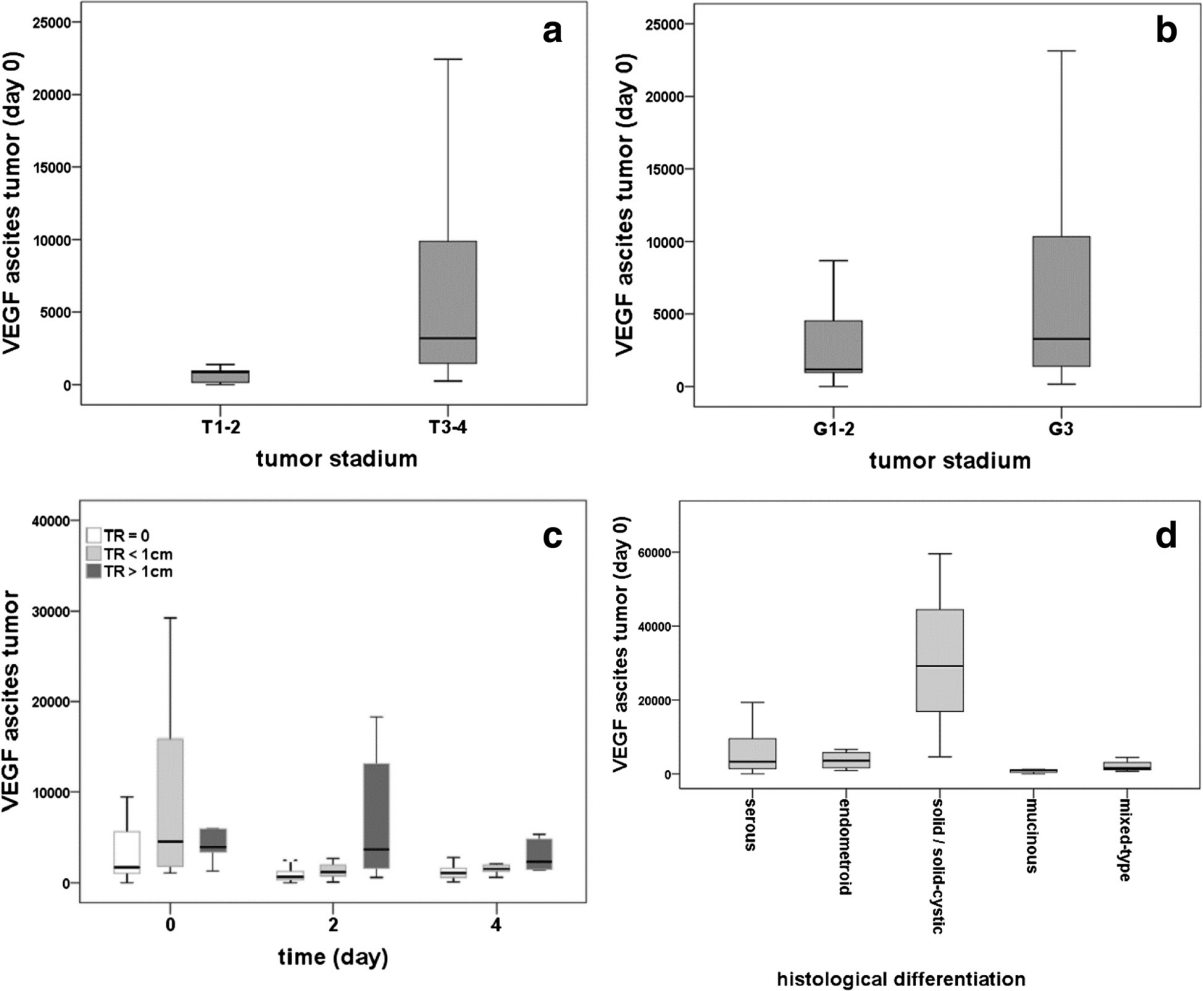
VEGF levels in ascites in dependency of tumor stage, grading, resection status and histological type. Comparison between VEGF levels (pg/ml) in the ascites samples of ovarian cancer patients between tumors of different sizes (T1-2 vs. T3-4). Statistically significant higher values in patients with T3-4 tumors were detected in comparison to T1-2 tumors (day 0) (*p* < 0.001) (**a**). Comparing VEGF levels in the ascites samples of ovarian cancer patients with well or moderately differentiated (G1/G2) and poorly differentiated (G3) tumors, we found a trend for higher values in patients with G3-tumors in comparison to G2/G1-tumors (day 0) (*p* = 0.061) (**b**). We revealed higher levels in patients with residual tumor after surgery (TR < 1 cm and TR > 1 cm) at day 0 in comparison to patients with no remaining tumor (TR 0). The differences between the group of patients with remaining tumor < 1 cm and the group with no residual tumor were significant at all days of measurement (all *p* < 0.05), and the differences between the group of patients with residual tumor > 1 cm and the group with no remaining tumor were significant on day 2 (*p* = 0.002) and on day 4 (*p* = 0.006) but not on day 0 (*p* = 0.063). There were no significant differences with regard to VEGF levels in ascites between the group of patients with remaining tumor < 1 cm and the group of patients with remaining tumor > 1 cm at any of the three days (all *p* > 0.05). A significant decrease of VEGF between day 0 and 2 could be detected in patients with no remaining tumor (*p* = 0.001) and patients with residual tumor < 1 cm (*p* = 0.009), but not in patients with remaining tumor > 1 cm (*p* = 0.499). (**c**). Analysis at day 0 of measurement revealed highest levels in the group of solid / solid-cystic tumors (**d**)

Since we supposed that the tumor itself might be the main source of VEGF, the concentration of VEGF was measured in the ascites at day 0, 2, and 4 after surgery. A significant decrease of VEGF was observed between day 0 and day 2 (*p* < 0.001) as well as between day 0 and day 4 (*p* < 0.001) with no difference between day 2 and day 4 (*p* = 0.320). Patients with T3-4 tumors had significantly higher levels of VEGF in ascites on day 0 in comparison to patients with T1-2 tumors (*p* < 0.001) (Fig. 3a); and there was a trend towards higher VEGF levels on day 0 in patients with poorly differentiated tumors (G3) in comparison to well or moderately differentiated tumors (G1/G2) (*p* = 0.061) (Fig. 3b). Measurement of VEGF levels in the ascites of patients with different resection status after surgery revealed higher levels in patients with residual tumor after surgery (TR <1 cm and TR >1 cm) in comparison to patients with no remaining tumor (TR 0) at all three measurement days (Fig. 3c). The differences between the group of patients with remaining tumor <1 cm and the group with no residual tumor were significant at all days of measurement (all *p* < 0.05), and the differences between the group of patients with residual tumor >1 cm and the group with no residual tumor were significant on day 2 (*p* = 0.002) and on day 4 (*p* = 0.006) but not on day 0 (*p* = 0.063). There were no significant differences with regard to VEGF levels in ascites between the group of patients with residual tumor <1 cm and the group of patients with residual tumor >1 cm at any of the three days (all *p* > 0.05). A significant decrease of VEGF levels between day 0 and 2 could be detected in patients with no remaining tumor (*p* = 0.001) and patients with remaining tumor <1 cm (*p* = 0.009), but not in patients with residual tumor >1 cm (*p* = 0.499). The comparison between VEGF values in the ascites of different histological types of ovarian cancer on day 0 of measurement revealed significant differences (*p* = 0.039) with highest levels observed in the group of cystic solid tumors (Fig. 3d).

### VE-cadherin and claudin 5 in the peritoneal vessels

**Fig. 4:**
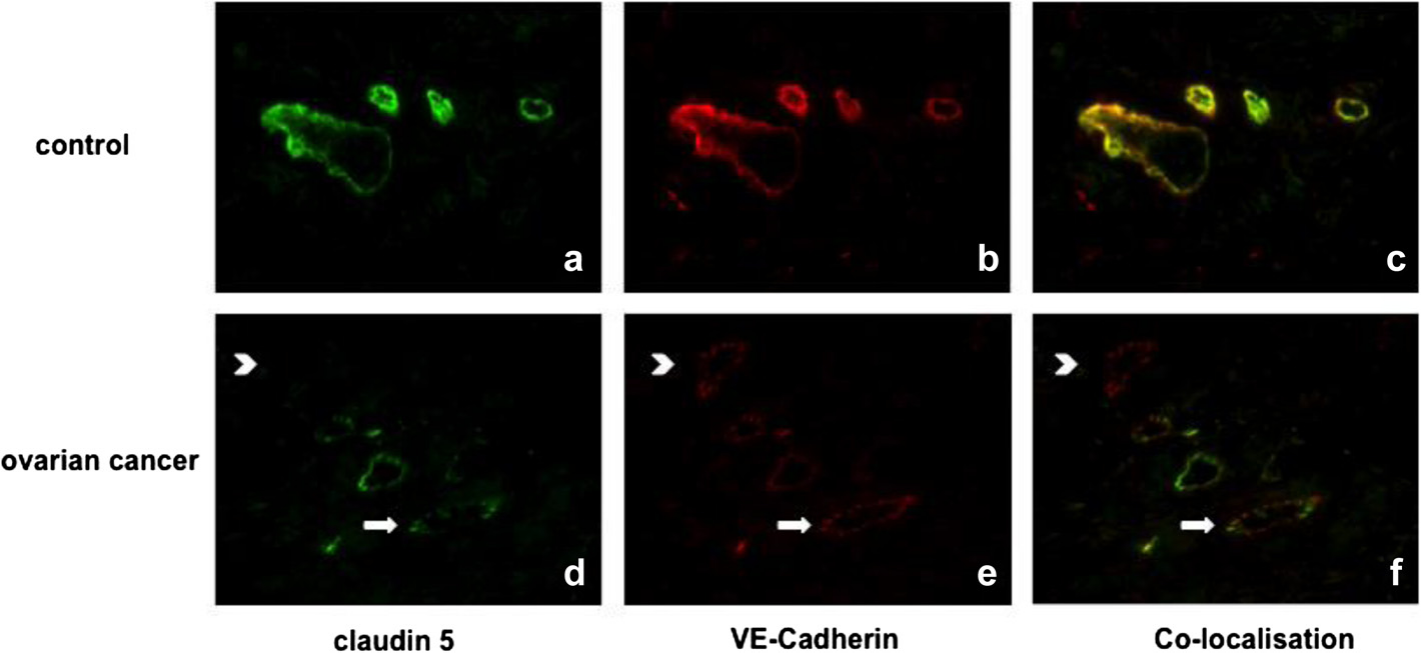
Claudin 5 and VE-Cadherin in the peritoneal vessels (ovarian cancer vs. control). Representative immunocytochemical staining of claudin 5 (green staning), VE-Cadherin (red staining) and their co-localisation (orange staining) in the peritoneal tissue (**a**-**c** control, **d**-**f** ovarian cancer). Note the general occurrence of both proteins in the endothelium of the same vessels in controls (**c**). In contrast in tumors the staining is generally weaker and discontinuously distributed (arrows). Some of the vessels lack one of the proteins (e.g. claudin 5) (arrowhead). Pictures are taken with fluorescence microscope under 40× magnification

Dual staining for VE-Cadherin or claudin 5 with CD 31 revealed that both proteins are localized to peritoneal vessels. Furthermore, dual staining for VE-Cadherin with claudin 5 clearly showed the co-existence of both proteins in the same vessel (Fig. 4a-c). In the peritoneal vasculature of tumor patients VE-Cadherin and claudin 5 protein are clearly down-regulated as indicated by an unevenly distributed staining (Fig. 4d-f). While in controls dual staining is generally found in nearly all vessels in tumor patients dual staining is generally weaker and varies a lot.

**Fig. 5:**
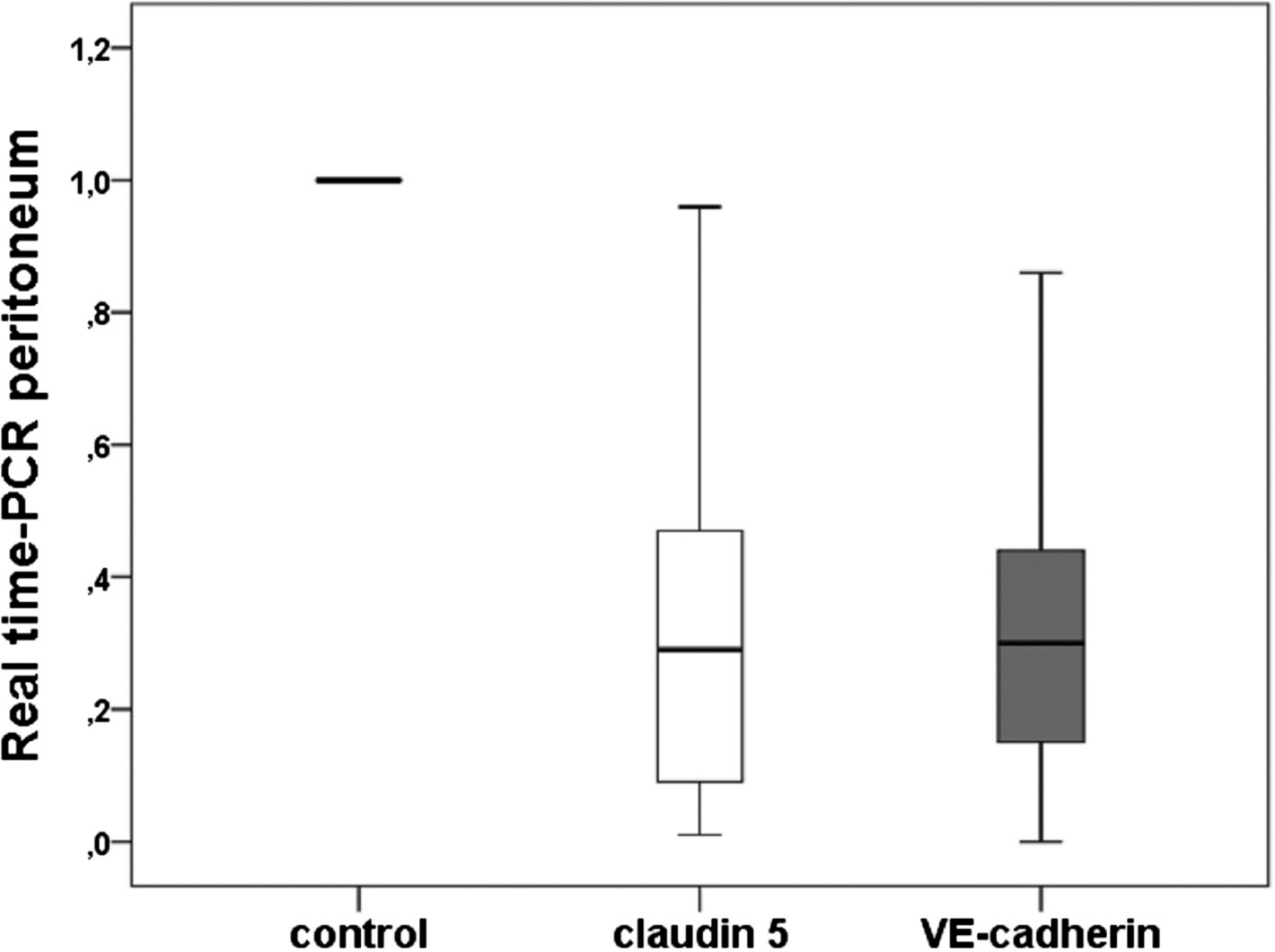
Expression of claudin 5 and VE-cadherin in the peritoneum (tumor vs. control). Real-time PCR-analysis of claudin 5 and VE-cadherin in the peritoneal vessels of ovarian cancer patients. We revealed statistically significant lower values of claudin 5- and VE-cadherin-expression in the peritoneal vessels of the tumor patients as compared to the healthy control group (both *p* < 0.001)

**Fig. 6:**
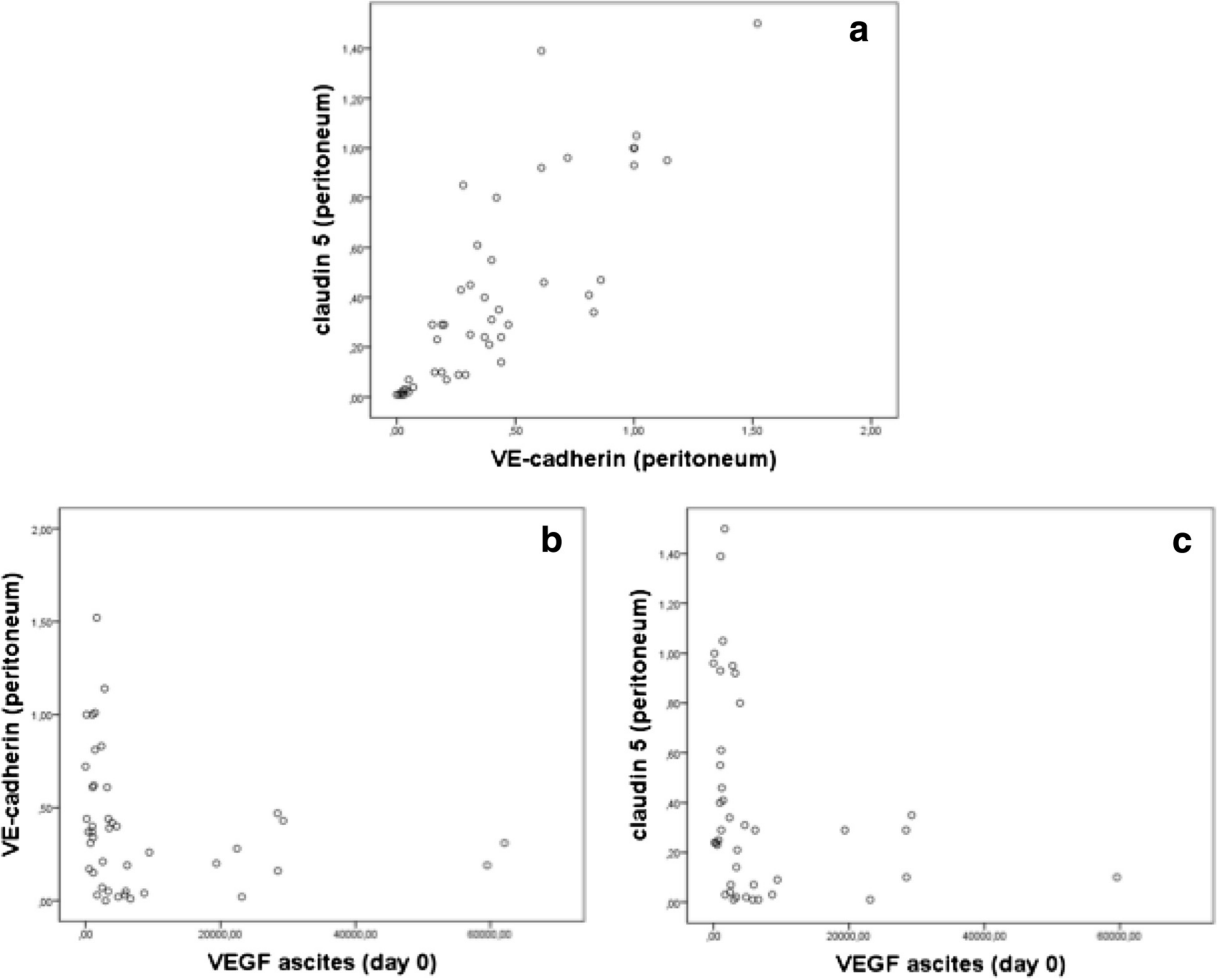
Correlation between adhesion protein expression (VE-cadherin and claudin 5) and VEGF in the peritoneum. Correlation between claudin 5 and VE-cadherin in the peritoneum of ovarian cancer patients. By plotting the values of real-time PCR-analysis against each other a significant linear correlation between claudin 5 and VE-cadherin was detected (r_S_ = 0.839, *p* < 0.001) (**a**). Plotting the VEGF expression against VE-cadherin or claudin 5 expression showed significant negative correlations (VE-c: r_S_ = −0,384, *p* = 0.012) (cl 5: r_S_ = −0.355, *p* = 0.021); the scatter plots indicate that there might be a hyperbolic function rather than a linear relationship in both cases (**b** and **c**)

As we had already demonstrated a significant reduction for claudin 5 levels in the peritoneal vessels of ovarian cancer patients as compared to the controls in a former study [9] we here performed an analysis on the level of gene expression. Real-time PCR revealed significant (*p* < 0.001) lower values of VE-cadherin as well as claudin 5 expression in the peritoneal vessels as compared to the healthy control group (Fig. 5). Plotting the values of VE-cadherin and claudin 5 in the peritoneal samples against each other we found a significant (r_S_ = 0.839, *p* < 0.001) positive correlation (Fig. 6a), suggesting that VE-cadherin and claudin 5 levels are regulated in a similar way. In contrast, VEGF levels in ascites at day 0 were negatively correlated to both VE-cadherin and claudin 5 expression in the peritoneal vessels (VE-c: r_S_ = −0,384, *p* = 0.012) (cl 5: r_S_ = −0.355, *p* = 0.021); the scatter plots indicate that there might be a hyperbolic function rather than a linear relationship in both cases (Figs. 6b and c).

Interestingly, analysis of VE-cadherin and claudin 5 expression in the peritoneal vessels in relation to various tumor characteristics (i.e. tumor stage, grading, histological type) revealed no significant differences.

### Analysis in human ovarian cancer cells and HUVEC in the co-culture-system

**Fig. 7:**
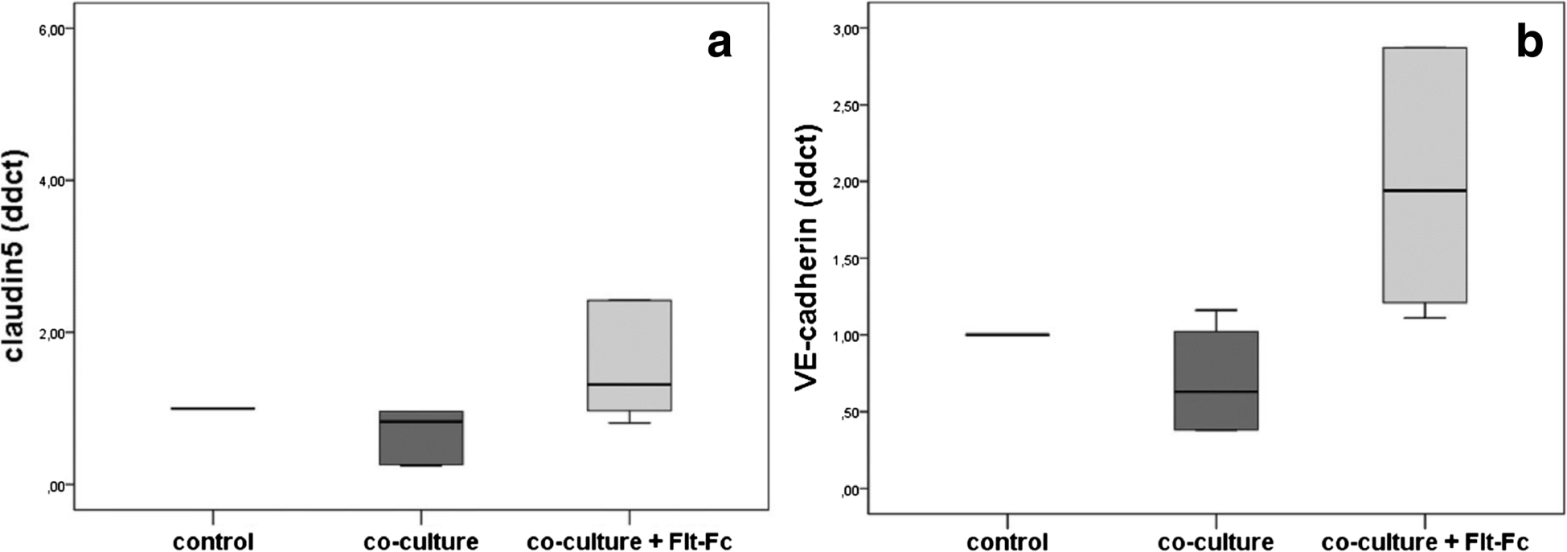
Changes of adhesion proteins co-cultured with HUVEC and ovarian cancer cells. Co-culture model of human ovarian cancer cells with HUVEC without (dark grey bar) and with (light grey bar) VEGF-inhibition via Flt1-Fc. Changes in claudin 5 and VE-cadherin were measured using Real-time PCR. VEGF inhibition revealed a significant increase of claudin 5 and VE-cadherin in comparison to the co-culture without inhibition (both *p* < 0.05) (**a** and **b**)

For the use of our human ovarian cancer cell line in co-culture we first of all confirmed that the cells express the VEGF gene and secrete VEGF in the culture medium by ELISA. Since it has been shown in vivo that the adhesion proteins VE-cadherin and claudin 5 are reduced in the peritoneal vessels of ovarian cancer patients, we measured the production of VE-cadherin and claudin 5 in HUVEC grown in the co-culture system in vitro. Confirming the results of our previous studies in a co-culture model with synthesized Ovcar-3 cells we found a significant (*p* < 0.05) decrease of claudin 5 in HUVEC co-cultured with our human ovarian cancer cells, which was prevented by simultaneous treatment with the VEGF-inhibitor Flt1-Fc. In addition, for VE-cadherin a comparable significant (*p* < 0.05) decrease in HUVEC co-cultured with our human ovarian cancer cells was detected, which was prevented by simultaneous treatment with the VEGF-inhibitor Flt1-Fc. Figures 7a and b illustrate these results.

## Discussion

Ascites as a sign of advanced tumor growth reduces the quality of life of ovarian cancer patients [8, 12]. Malignant ascites is known to arise from both tumor surface and tumor peritoneum [13]. This metastatic pattern is dependent on establishing angiogenesis at the newly seeded site [14]. Here VEGF plays an important role by promoting neovascularization and enhancing vascular permeability, thus leading to intraabdominal tumor growth, tumor spread, peritoneal carcinomatosis and ascites formation [15]. Previously we demonstrated a potential mechanism of ascites production in serous ovarian cancer patients by VEGF affecting tight junctions in the peritoneal vasculature [9]. In the current study we demonstrate for the first time, that VEGFA, known as the most important angiogenic factor, is differentially expressed in dependency of tumor stage, grading, histological type and surgical resection status, with highest expression in advanced tumor stage (T3-4), poorly differentiated tumors (G3), solid tumors and after resection with residual macroscopic disease (TR). In addition, we demonstrate that under the influence of tumor derived VEGF different cell adhesion types (tight and adherens junctions) interact in the peritoneal vasculature to regulate endothelial permeability. The down regulation of adherens junctions (VE-cadherin) and subsequent suppression of tight junctions (claudin 5) by VEGF, which is accumulated in serum and ascites, may account for the massive loss of fluid into the abdominal cavity in advanced ovarian cancer.

The current study confirmed our previously obtained preliminary observations obtained on 10 tumor patients [9]. In consistency with the former study, VEGF was significantly increased in the serum as well as in ascites of preoperative ovarian cancer patients as compared to healthy controls. These findings are consistent with other investigations of VEGF levels in ovarian cancer in comparison to benign cystadenomas [16–18]. VEGF is known to be expressed to a great extent in the majority of ovarian cancers and its risen serum levels and tumor levels have been found to be independent markers of poor clinical outcome [19]. In addition, elevated VEGF levels in malignant ascitic fluids were reported to be of prognostic significance [20]. Our results indicate that VEGF does not only play a local role (in the abdomen via regulation of endothelial permeability and ascites formation) but also a systemic role (blood) in ovarian cancer patients. VEGF secreted into the bloodstream may possibly interact on the vasculature of other serous tissue, such as pleura. This may explain that sometimes pleural effusion is observed in ovarian cancer patients without any tumor affection of the pleura [21].

Consistent with our previous study, measurement of VEGF in the serum preoperative, on day 2 and 4 after surgery revealed a significant decrease of VEGF levels 2 days after surgery and a significant increase at day 4 [9]. The effect of tumor reduction via surgery is clearly reflected by a significant decrease of the VEGF amount in the serum and ascites two days after surgery. However, the increase of serum VEGF at day 4 after surgery appears to be paradox, especially since this increase was not observed in the ascites samples. This finding may be explained by the fact, that after surgery wound healing and tissue regeneration processes occur which are associated with increase in angiogenesis induced by VEGF that is now synthesized by other tissue and released systemically [22–24].

As a main focus of this study, the potential differences of permeability regulation in dependency of varying tumor stage, grading, histological ovarian cancer subtypes and resection status after surgery were investigated. In this study we detected for the first time differences of VEGF synthesis in various histological and biological tumor types. Regarding the histological type, ascites samples revealed significant highest VEGF levels in patients with solid tumors which are associated with worse clinical outcome and more aggressiveness in tumor growth [25] as compared to the group of serous, endometrioid, mucinous and mixed-type tumors. Moreover, we revealed higher values of VEGF levels in serum and ascites samples of patients with ovarian cancer of advanced tumor stages (T3-4 tumors) indicating that the VEGF level correlates positively with the total tumor mass. VEGF is known to be the most important angiogenic factor promoting tumor growth through stimulation of angiogenesis [26]. Through this mechanism tumor-derived VEGF enhances the peritoneal vasculature permeability leading to the formation of malignant ascites, tumor spread, dissemination and growth as well as formation of peritoneal metastasis. Bryne et al. reported that enforced expression of VEGF by ovarian cancer cells dramatically reduced the time to onset of ascites formation [27]. This is in line with the clinical observation that ovarian cancer types of early stage often do not present with ascites formation [3]. It may be hypothesized that there exists a critical tumor mass which produces enough VEGF for ascites production. This hypothesis is further supported by our finding that VEGF levels tended to be higher in ascites of patients with poorly differentiated tumors (G3) as compared to well or moderately differentiated tumors (G1/G2). Poorly differentiated tumors are growing faster according to high tumor cell proliferation. These patients with poorly differentiated tumors are often diagnosed in advanced tumor stages i.e. with high tumor load. The aggressiveness of these tumors leading to increased VEGF may facilitate local metastasis in the peritoneum by induction of angiogenesis so that the tumor may be early connected and spread via the vascular system [17, 28, 29].

Our results concerning the different tumor resection status after surgery (no residual tumor, remaining tumor <1 cm or >1 cm) further support the fact that the total tumor mass is critical for VEGF secretion. Here, we found a positive correlation between tumor load and VEGF levels both in serum and ascites samples with higher values of VEGF in the patient group with residual tumor in comparison to those being successfully operated tumor free. This difference in VEGF levels could be detected for all days of measurement, before (day 0) as well as day 2 and 4 after surgery. Highest levels of VEGF were detected in the group of patients with the largest residual tumor (TR >1 cm). Concerning clinical systemic treatment guidelines of ovarian cancer, these results underline the effectiveness of anti-angiogenic therapies such as VEGF-antibody therapies in patients with advanced tumor stages and remaining tumor [30]. Since the VEGF levels of patients with a final residual tumor after operation are already elevated in serum and ascites before surgery (day 0) in comparison to those with no postoperative residual tumor, the question arises whether differences in VEGF expression could predict which of the patients will be successfully operated tumor free.

Ascites production due to increased peritoneal permeability appears to be dependent on suppression of cell adhesion proteins [9]. For instance, inhibition of VE-cadherin leads to a dissolution of tight junctions and thus secondary to increased vascular permeability [11]. At the same time a VEGF-dependent regulation of the junctional protein claudin 5 with a consecutive increase of permeability was detected in our previous studies in an in vitro corpus luteum model [31] and in serous ovarian cancer [9]. Based on these data, we assumed a functional role of VEGF-dependent regulation of VE-cadherin and claudin 5 concerning regulation of endothelial permeability also in the peritoneal tissue of ovarian cancer patients. In order to investigate this presumption, a new co-culture system of *human* ovarian cancer cells (to simulate the conditions in vivo) and endothelial cells (HUVEC) was used. As expected, due to VEGF produced by the ovarian cancer cells, endothelial cells co-cultured with our human ovarian cancer cells presented with significant lower levels of VE-cadherin and claudin 5. This effect was prevented by simultaneous treatment with the VEGF-inhibitor Flt1-Fc validating the functional dependence between VEGF and the adhesion proteins. For the in vivo situation we detected VE-cadherin being co-localised with claudin 5 in the majority of the peritoneal vessels. Expression of both molecules was in general significantly decreased in the peritoneum of ovarian cancer patients. In addition, some of the vessels or parts of the peritoneal endothelium lack one of these proteins completely indicating the disturbance of normal cell adhesion resulting in increased permeability and ascites formation. Regarding the interaction of VE-Cadherin and claudin 5 we found a significant positive correlation for expression of both adhesion proteins, indicating that both membrane proteins are regulated similarly and that they may interact with each other, as described by other studies [10].

Since we proved in vitro the functional influence of VEGF on expression of cell adhesion proteins and we found in situ higher VEGF levels in solid tumors, T3-4 and G3 tumors, we hypothesized that in these subtypes VE-cadherin and claudin 5 are significantly more suppressed as compared to the other subtypes. However, we did not find any differences in gene expression between different histological subtypes, tumor stages or grading. It was furthermore suspected that VE-cadherin and claudin 5 were negatively correlated to the VEGF levels. Indeed, our analyses revealed significant negative correlations between VEGF levels and the expression of adhesion proteins (both VE-cadherin and claudin 5); however, the scatter plots indicated a hyperbolic function rather than a linear relationship. This indicates that as soon as a certain threshold value of VEGF is reached gene expression of the VE-cadherin and claudin 5 is switched off and no further increase of VEGF is necessary to have a further suppressive influence on these membrane proteins.

Overall, our findings suggest that ovarian cancer cells produce VEGF in order to first induce angiogenesis to allow tumor growth and second increase endothelial permeability via suppression of VE-cadherin and subsequent claudin 5 in the peritoneal vasculature, which finally induces ascites and thereby facilitates dissemination of cancer cells in the abdominal cavity. Since aggressive and advanced tumors synthesise significantly more VEGF, dissemination of cancer cells may be facilitated and thus metastases may develop earlier than in less aggressive tumors, which is in accordance with the poor prognosis of the aggressive tumor subtypes.

## Conclusions

In summary, our results demonstrate that VEGF plays an important role in pathological hyperpermeability and ascites formation of *human* ovarian cancer via down-regulation of a cascade of adhesion proteins (VE-cadherin and subsequent claudin 5). Increased VEGF levels were associated with histologic solid tumors, high (T3-4) tumor load, poor tumor differentiation and tumor rest after surgery in serum and/or ascites samples. Thus, it is assumed that advanced ovarian cancer types are characterized by early dysregulation of vascular permeability leading to ascites and tumor spread. These patients may mostly benefit from therapeutic VEGF inhibition. In addition, measurement of early post-surgical serum VEGF levels may predict which patient will mostly profit from an adjuvant VEGF-antibody therapy.

## Methods

### Patients

The tumor- and peritoneal tissue as well as serum and ascites samples used for the experiments were collected from patients undergoing laparotomy for ovarian cancer. Tumor and peritoneal tissue samples were collected during surgery in liquid nitrogen for further RNA-isolation and in 3.5–3.7 % formaldehyde (Fischar, Saarbrücken, Germany) for immunohistochemistry. In addition, serum and ascites samples of all subjects were collected before surgery (day 0) as well as two days (day 2) and four days (day 4) after surgery. As controls, we used tissue samples of patients undergoing surgery for benign reasons such as uterine myoma or uterine prolapse.

The collection and use of human tissue, ascites and serum was institutionally approved by the Ethics Committee of the University of Ulm and the patients had given their informed consent.

### Preparation of tissue for H&E staining and Immunhistochemistry

The tumor- and peritoneal tissue samples were fixed in 3.5–3.7 % formaldehyde (Fischar, Saarbrücken, Germany) for 24 h and then incubated in 70 % ethanol at room temperature overnight. Afterwards the tissue was dehydrated for 45 min at 40 °C in ascending concentrations of ethanol, then 2 x 45 min of xylol and in the end 2 x 60 min in Paraplast Plus (Tissue Embedding Medium, Leica, Richmond, USA).

### Morphological characterization of ovarian cancer

Consecutive sections stained for hematoxylin and eosin were used to classify the ovarian cancer and peritoneal tissue. Therefore, the embedded ovaries were serially sectioned, and tissue sections (3 μm) were placed onto SuperFrost Plus slides (VWR, Leuven, Belgium). Tissue sections were dewaxed in xylene, rehydrated in descending concentrations of ethanol, washed in distilled water, and stained with hematoxylin (Mayer’s hemalum solution, Merck, Darmstadt, Germany) for 3 min, followed by a rinsing with hydrochloric acid 0.1 % and by a wash in water and acetic alcohol before staining with eosin (Eosin Y-solution 0.5 % aqueous, Merck, Darmstadt, Germany) for 3 min. After dehydrating in ascending concentrations of ethanol and xylene, sections were mounted in Eukitt (Kindler, Freiburg, Germany).

The final tumor staging (TNM classification) was obtained from the report of the institutional pathologist and documented for each ovarian cancer patient for later analysis. Data on the intraoperative resection status of each tumor patient were obtained from the operation report.

### Immunohistochemistry dual staining (VE-cadherin and claudin 5)

Immunofluorescence double-staining was performed using the TSA-Kit (Perkin Elmer, Boston, USA). Sections of paraffin-embedded peritoneal tissue were dewaxed and rehydrated using xylol and ethanol, respectively, and transferred to TN-buffer. Slides were incubated in Target Retrieval Solution pH 9 (1:10) at 95 °C for 30 min in the water bath, left in the hot buffer for another 20 min, cooled down and transferred to TN-buffer for 5 min. Endogenous peroxidase was quenched for 30 min in 180 ml methanol + 20 ml hydrogen peroxide (30 %), and slides were again transferred to TN-buffer for 5 min. After pre-incubation with TNB for 30 min, the slides were incubated with the mouse anti-human CD 31 antibody (Dako, Hamburg, Germany; 1:30 dilution) over night at 4 °C to prove the co-localization to the endothelial compartment in the further step. The slides were then washed in TN-buffer with 0.1 % Tween for 3 × 5 min, followed by incubation with biotinylated rabbit anti mouse secondary antibody (Dako, Hamburg, Germany; dilution 1:750) for 45 min. Washing in TN-buffer + Tween and incubation with SA-horse-radish peroxidase (HRP) and Fluoresceine tyramide was performed according to the instructions of the manufacturer. Incubation with the second primary antibody, the antibody mouse anti-human VE-cadherin (Merck Millipore, Darmstadt, Germany; dilution 1:20) or the antibody mouse anti-human claudin 5 (Invitrogen, Carlsbad, USA; dilution 1:100), overnight at 4 °C was followed by washing, incubation with biotinylated secondary antibody, SA-HRP and TMR tyramide as described above. Mounting was performed with Roti-Mount FlourCare. In order to prove co-localization of VE-cadherin with claudin 5 in the same vessel double-staining was performed as described above using claudin 5 instead of CD 31. Pictures were taken with Keyence fluorescence microscope BZ-9000 under 40× magnification.

### Quantification of VEGF by ELISA (serum and ascites)

The analysis of our study was focused on the most important pro-angiogenic factor VEGF-A (splice variant 165). In order to quantify the secreted amount of VEGF, a quantitative VEGF immunoassay was performed according to the manufacturer’s protocol (R&D Systems, Minneapolis, USA). Briefly, 100 μl of control, sample or standard was added to 100 μl assay diluent. After 2 h incubation at room temperature, the samples were washed three times. Then 200 μl VEGF conjugate was added for 2 h and the samples were washed again. After incubation with 200 μl substrate solution for 25 min, 50 μl stop solution was added and the optical density was measured at 450 nm (Elisareader Sunrise, Tecan, Männedorf, Switzerland).

### Endothelial cell isolation and culture

The collection of human primary cells was institutionally approved after favorable ethical review. Human umbilical cords were rinsed with water and disinfected with isoseptol. Under sterile conditions the ends of the cords were cut and into each end a flexible tube was inserted and fixed with a cable tie. The umbilical veins were rinsed with phosphate buffered saline (1 x PBS). One end was clamped and from the other end the vein was filled with type 1 collagenase (1 mg/ml; Sigma, Saint Louis, USA) to detach the endothelial cells. Subsequently, the second end was clamped and the cords were incubated in a water bath at 37 °C for 15 min.

Human umbilical vein endothelial cells (HUVEC) were collected and mixed 1:1 with endothelial cell growth medium (Promocell C-22010, Heidelberg, Germany) + 10 % fetal calf serum (FCS) with 1 % penicillin/streptomycin (Gibco by Life Technologies, Carlsbad, USA). After centrifugation for 5 min at 1200 rpm the supernatant was discarded and the pellet was resuspended in culture medium. The HUVEC were seeded either in primaria tissue flasks (25 cm^2^) or in 6-well plates and incubated at 37 °C in 5 % CO_2_.

### Cell culture for human ovarian cancer cells

In order to work with *human* ovarian cancer cells we used ovarian cancer cell lines of our department, which have been established before by Möbus et al. [32].

For our experiments the human ovarian cancer cells were cultivated in DMEM with GlutaMax (Gibco by Life Technologies, USA) with 1 % Penicillin/Streptomycin 10 % FCS (FBS superior, Biochrom, Berlin, Deutschland). Media were changed twice a week.

### Co-culture of ovarian cancer cells and HUVEC

**Fig. 8:**
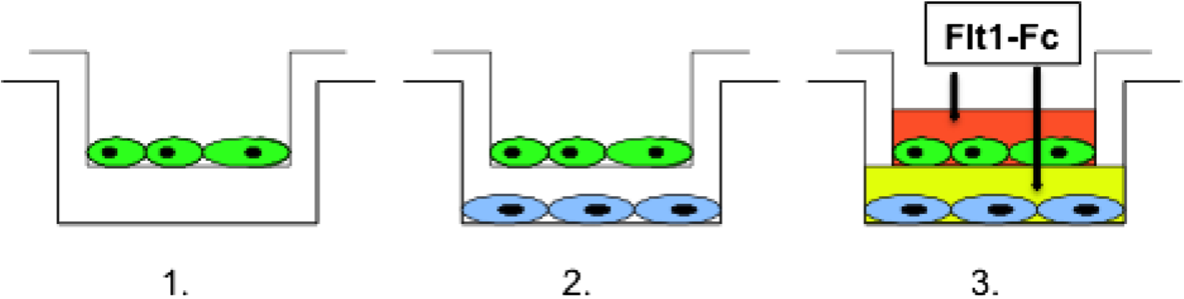
Co-culture of ovarian cancer cells and HUVEC. Schematic drawing illustrating the co-culture model of HUVEC (green) separated from the human ovarian cancer cells (blue) by a permeable membrane (pore size 0.4 μm). Treatments involved a negative control with HUVEC and an empty well (**1.**), co-culture with ovarian cancer cells (**2.**) and VEGF-inhibition by addition of Flt1-Fc to both cell compartments (**3.**) (see also Herr et al. 2012)

For our co-culture experiments we used our well-established co-culture model by Herr et al. [9]. Instead of treatment with conventional ovarian cancer cell-lines we used a co-culture system with HUVEC and our *human* ovarian cancer cell lines. Treatment involved first a negative control with HUVEC only, second the co-culture with ovarian cancer cells and HUVEC and third VEGF-inhibition by addition of Flt1-Fc (VEGF-Receptor-1 (Flt-1)/Fc Chimera, Mouse, recombinant; Steinheim, Germany) to both cell compartments. Figure 8 illustrates the co-culture setting.

HUVEC (2x10^5^ per well) were seeded onto 6-well plates and grown to 90–100 % confluency. On the same day of culture, human ovarian cancer cells (2 x 10^5^ cells) were transferred to cell culture inserts (0.4 μm pore size, Becton Dickinson Nr. 353090). On day 2, cell culture inserts with human ovarian cancer cells were inserted into the 6-well plates with HUVECs in culture. Endothelial cells were treated with 200 ng and ovarian cancer cells with 300 ng Flt1-Fc. In this setting, molecules secreted by ovarian cancer cells follow the concentration gradient between the two different media such that the HUVEC can be stimulated.

### Preparation of tissue for RNA-isolation

50–100 mg of the pulverized tumor- and peritoneal tissue was mixed with 1 ml Trizol, incubated at room temperature for 5 min and mixed with 200 μl chloroform. After 3–5 min incubation at room temperature and centrifugation the aqueous phase was mixed with 500 μl isopropanol and incubated for 5–15 min at 4 °C. After centrifugation the pellet was washed twice with 1 ml 75 % ethanol in DEPC-H_2_O and centrifuged for another 10 min at 4 °C. Then the pellet was dispensed in RNA-free H_2_O for 10 min at 55 °C. RNA was measured at the spectrophotometer (Nanodrop 2000; Peqlab, Erlangen, Germany) and stored at −80 °C.

### RNA-Isolation

Total RNA from HUVEC, human ovarian cancer cells and ovarian cancer tissue was extracted from cells with peqGOLD TriFast reagent (Peqlab, Erlangen, Germany). The RNA product was quantified by absorbance at 260 nm and total RNA (1.0 μg) was reverse transcribed into cDNA using the cDNA High capacity Reverse Transcription kit (Applied Biosystems, Foster City, USA) according to the manufactures’s instructions.

### Real-time-PCR

For quantification of VE-Cadherin and Claudin 5 expression Taqman Gene Expression Assays (VE-Cadherin: Hs00174344_m1; Claudin 5: Hs00533949_s1; Applied Biosystems by life technologies, Darmstadt, Germany) were utilized according to the manufacturer’s instructions using TaqMan™ Universal PCR Master Mix (Applied Biosystems by life technologies, Darmstadt, Germany). Amplification and detection of specific products was performed with Vii A 7™ Real-Time PCR System (Applied Biosystems by life technologies, Darmstadt, Germany). The quantity of cDNA was normalised to the quantity of ß2-Microglobulin cDNA in each sample. Calculation of relative gene expression was performed using the comparative 2^-ΔΔ^CT method.

### Statistics

Statistical analysis were performed using SPSS for Windows version 21.0. The data distribution of most variables were significantly different from normal distributions; thus non-parametric statistical procedures were used for all analyses presented here. Independent groups were compared using Kruskal-Wallis and Mann–Whitney U tests, while data obtained for samples of the same patients on different days were analysed with Friedman tests and Wilcoxon matched pairs tests for related data. Comparisons among groups are illustrated using Box-and-Whisker plots, where the horizontal line inside the box represents the median and the box indicates the interquartile range (IQR; the middle 50 % of scores). The ends of the whiskers denote the lowest and highest values still within 1.5 IQR of the lower and upper quartile (i.e. the lower and upper end of the box), respectively. If there are no values more than 1.5 IQR below the lower or above the upper quartile (i.e. outliers), the ends of the whiskers denote minimum and maximum of the data. Correlation tests were performed using Spearman’s rank correlation coefficient. All statistical tests were two-tailed and p-values below 0.05 were considered to be statistically significant.
